# Pathogenicity Levels of Colombian Strains of *Candida auris* and Brazilian Strains of *Candida haemulonii* Species Complex in Both Murine and *Galleria mellonella* Experimental Models

**DOI:** 10.3390/jof6030104

**Published:** 2020-07-10

**Authors:** Julián E. Muñoz, Laura M. Ramirez, Lucas dos Santos Dias, Laura A. Rivas, Lívia S. Ramos, André L. S. Santos, Carlos P. Taborda, Claudia M. Parra-Giraldo

**Affiliations:** 1 MICROS Group, Medicine Traslacional Institute, School of Medicine and Health Sciences, Universidad del Rosario, Bogotá, D.C. 111221, Colombia; juliane.munoz@urosario.edu.co; 2 Unidad de Proteómica y Micosis Humanas, Grupo de Enfermedades Infecciosas, Departamento de Microbiología, Facultad de Ciencias, Pontificia Universidad Javeriana, Bogotá, D.C. 110231, Colombia; lramirez-r@javeriana.edu.co (L.M.R.); lrivas@usp.br (L.A.R.); 3 Department of Microbiology, Biomedical Sciences Institute, University of São Paulo (USP), São Paulo, SP 05508-060, Brazil; dossantosdia@wisc.edu (L.d.S.D.); taborda@usp.br (C.P.T.); 4 Laboratório de Estudos Avançados de Microrganismos Emergentes e Resistentes, Departamento de Microbiologia Geral, Instituto de Microbiologia Paulo de Góes (IMPG), Centro de Ciências da Saúde (CCS), Universidade Federal do Rio de Janeiro (UFRJ), Rio de Janeiro 21941-901, Brazil; liviaramos2@yahoo.com.br (L.S.R.); andre@micro.ufrj.br (A.L.S.S.); 5 Laboratory of Medical Mycology-LIM53/IMTSP, University of São Paulo (USP), São Paulo, SP 05508-060, Brazil

**Keywords:** *Candida auris*, *Candida haemulonii* species complex, virulence, candidiasis, biofilm, *Galleria mellonella*

## Abstract

*Candida auris* and *Candida haemulonii* complex (*C. haemulonii, C. haemulonii* var. *vulnera* and *C. duobushaemulonii*) are phylogenetically related species that share some physiological features and habits. In the present study, we compared the virulence of these yeast species using two different experimental models: (i) *Galleria mellonella* larvae to evaluate the survival rate, fungal burden, histopathology and phagocytosis index and (ii) BALB/c mice to evaluate the survival. In addition, the fungal capacity to form biofilm over an inert surface was analyzed. Our results showed that in both experimental models, the animal survival rate was lower when infected with *C. auris* strains than the *C. haemulonii* species complex. The hemocytes of *G. mellonella* showed a significantly reduced ability to phagocytize the most virulent strains forming the *C. haemulonii* species complex. Interestingly, for *C. auris*, it was impossible to measure the phagocytosis index due to a general lysis of the hemocytes. Moreover, it was observed a greater capability of biofilm formation by *C. auris* compared to *C. haemulonii* species complex. In conclusion, we observed that *C. auris* and *C.* *haemulonii* complex have different levels of pathogenicity in the experimental models employed in the present study.

## 1. Introduction

*Candida auris* and *Candida haemulonii* complex (*C. haemulonii, C. haemulonii* var. *vulnera* and *C. duobushaemulonii*) are phylogenetic related species, being the first one constantly related with healthcare-related infectious diseases (HID). These yeast species present ubiquitously distribution and inherently multidrug-resistance profile, especially against polyenes (e.g., amphotericin B) and azoles (e.g., fluconazole, itraconazole and voriconazole) [1].

*Candida auris* was isolated for the first time from the external auditory canal of a female patient in Japan in 2009 and in 15 South Korean patients in the same year [2,3]. It is a multidrug-resistant agent mainly related with HID, showing high rate of mortality and diversity of susceptibility profiles. This pathogenic yeast was reported in countries as Venezuela (2012), India (2013), South Africa and Kuwait (2014), Colombia (2015), Spain (2016), United States of America (2016), Panama (2017), United Kingdom (2017) and it continues to expand around the world [4,5,6,7,8,9,10,11].

Among its mainly features, *C. auris* is able to colonize a variety of surfaces, from inert materials to multiple body parts, presenting a large range of clinical manifestations [12,13]; (C) *auris* infection treatment can be tough due to the high resistance index that presents to most antifungals. One of the best therapeutic options to control a *C. auris* infection is the use of echinocandins, however, cases of resistance to this antifungal have already been reported [13,14]; (C) *auris* also stands out for its versatility to adapt to saline environments and, therefore, to resist desiccation. Recently, it was described that the hospital environment is an important risk factor in the process of *C. auris* transmission, since in many cases the intrahospital disinfection process is not enough to eradicate this fungus from the surfaces, which facilitates the transmission to other patients [15]. These patients are generally those with serious underlying medical conditions, including hematological malignancies and other conditions resulting in immunosuppression [6,13,16]. Therefore, it is urgent and necessary to design efficient antifungal strategies that help to prevent possible deaths caused by *C. auris* infection [17].

*Candida haemulonii* was found for the first time inside the intestine of a blue-striped fish (*Haemulon scirus*) in 1962. The first description of human infection was reported in 1984 from the blood of a patient, who died of renal failure despite of therapy with amphotericin B and flucytosine [18]. This yeast can present different clinical manifestations from onychomycosis to a deep mycosis in neonates in intensive care unit [19]. In fact, *C. haemulonii* is a complex formed by three genotypic differentiated species (*C. haemulonii, C. haemulonii* var. *vulnera* and *C. duobushaemulonii*). Candidiasis by *C. haemulonii* is rare compared to other species as *C. albicans*. However, it is important to note that these cases of candidiasis are severe due to the (multi) drug–resistance profile. Actually, resistance to fluconazole and amphotericin B has been defined, as well as strains presenting resistant to echinocandins were also described [14,20]. However, different susceptibility assays have demonstrated that *C. haemulonii* can be vulnerable to micafungin and caspofungin [21].

Since 2006, a close phylogenetic relationship had been observed between strains of *C. haemulonii* complex and *C. auris* that could only be resolved up to 2009 with the work of Satoh and collaborators [3] and later ratified by Cendejas-Bueno et al. 2012 [22], where they could differentiate the *C. haemulonii* complex from *C. auris*. The aim of the present work is to compare the virulence of *C. auris* versus the three species forming the C. *haemulonii* complex by means of in vitro and in vivo assays using *Galleria mellonella* larvae and BALB/c mice as models.

## 2. Materials and Methods

### 2.1. Microorganisms

Five strains of *Candida auris* (Ca432, Ca446, Ca386, Ca885 and Ca881) isolated from 2 different regions of Colombia and belonged to the Latin American clade IV, were selected to the present study. The clinical isolates Ca432, Ca446 and Ca386 of *C. auris* were obtained from Proteomic and Human Mycosis Investigation Unit, of Universidad Javeriana. Ca885 and Ca881 strains were obtained from Centro de Investigaciones Microbiológicas del Cesar (CIMCE). Six strains of the *C. haemulonii* complex distributed as *C. duobushaemulonii* (LIPCh1, LIPCh6) *C. haemulonii* (LIPCh3, LIPCh7, LIPCh12) and *C. haemulonii* var. *vulnera* (LIPCh11) [14] were obtained from Laboratório de Estudos Avançados de Microrganismos Emergentes e Resistentes of Universidade Federal do Rio de Janeiro (UFRJ). Strain identification was carried out by molecular and MALDI-TOF tests to verify the received material, as it is shown in Table 1. Standard strains of *C. albicans* (SC5314 and ATCC10231) were also included as controls. All strains were maintained at −80 °C. To perform the experiments, the fungi were subcultured in Sabouraud dextrose broth (Becton, Dickinson and Company; Sparks, NV, USA) at 37 °C for 24 h at 150 rpm before each assay. Entirely yeast panel utilized in this study were maintained and cultivate under the same conditions. However, the yeasts growth rates were different; *C*. *auris* duplicated after 2 h, while yeasts of the *C. haemulonii* complex duplicated after 3 h. To prepare the inoculum, the fungal cells were centrifuged and resuspended in a saline solution and, subsequently, quantified using a hemocytometer.

### 2.2. Galleria mellonella

The larvae were obtained from Scientia S.A. (Cali, Colombia) and maintained at 4 °C in dark conditions until the inoculation day. Before infection, decontamination of larvae was carried out using two different beakers, one with 250 mL of sterile distilled water and another with 250 mL of 0.1% sodium hypochlorite, in constant agitation for 30 s. Groups with 10 larvae each were placed in petri plates at 37 °C constantly to continue with the infection procedure.

### 2.3. Galleria mellonella Infection

The larvae were infected with 10 μL of the inoculum (5 × 10^4^ cells/larva) using insulin syringe in the proleg. After being inoculated, the larvae were placed at 37 °C in appropriate containers for their maintenance. Each group was composed with 10 larvae, with a proximally weight between 275–330 mg.

### 2.4. Survival in Galleria mellonella

Twelve groups with 10 larvae each were selected and then infected with *C. auris,* C. *haemulonii* species complex or *C. albicans* SC5314 (control group). Death of the larvae was scored daily for 10 days. Results were analyzed statistically.

### 2.5. Fungal Burden in Galleria mellonella

Different groups of infected larvae were sacrificed with 2 h or 3 days after infection. The whole larvae were macerated with tips in microtubes with 1 mL of 0.9% NaCl. After that, serial dilutions (1/10, 1/100 and 1/1000) were made, and 100 μL of each dilution were seeded on Sabouraud agar. The plates were incubated at 37 °C for 48 h, and colony forming units (CFU) were counted. Each experiment was performed in triplicate.

### 2.6. Histopathology of Galleria mellonella

Infected larvae were sacrificed after 3 h in the case of *C. auris* or after 3 days in the case of C. *haemulonii* species complex. Immediately, after slaughter the larvae were fixed in 10% buffered formalin and embedded in paraffin for sectioning. Slices were stained with periodic acid–Schiff (PAS) and examined microscopically at 10× magnification (Optiphot-2; Nikon, Tokyo, Japan).

### 2.7. Yeast Phagocytic Capacity of Galleria Mellonella hemocytes

For this test, the larvae infected with *C. auris* were kept alive for 3 h and in the case of *C. haemulonii* were kept for 3 days. Subsequently, a small incision was made in the pseudo-leg to obtain a drop of hemolymph, which was smeared on glass slides. After drying at room temperature, Wright dye was used to stain the samples and then they were observed under a microscope with a 100× magnification. In each slide 100 hemocytes were counted, then the hemocytes that had yeast inside and those that were empty were also counted with the aim to obtain the phagocytic percentage.

### 2.8. BALB/c Mice

Thirty-six isogenic females of BALB/c mice (6–8 weeks old) were bred at the University of São Paulo (São Paulo, Brazil) in an animal facility under specific pathogen-free conditions, constant temperature, light and dark cycles 12/12 h, ad libitum feeding and under the supervision of a single person trained and specialized in laboratory animal care. Procedures involving animals and their care were conducted according to the local ethics committee and international rules. All experiments were approved by the Institutional Animal Care and Use Committee of Institute of Biomedical Sciences (ICB), in the University of São Paulo (042-127-02, 12 April 2012).

### 2.9. Survival in BALB/c Mice

Six female BALB/c mice per group were immunosuppressed intraperitoneally with 2 doses of cyclophosphamide; 96 h and 24 h before intravenous infection with 1 × 10^4^ yeasts of *C. auris* (Ca432, Ca446), *C. haemulonii* (Ch3, Ch11) or *C. albicans* (ATCC 10231) as positive control. Since these strains of *C. auris* and *C. haemulonii* complex were the most virulent in the *G. mellonella* survival test, they were selected to carry out the present assay. The negative control group received phosphate-buffered saline (PBS). Three days after infection, another dose of cyclophosphamide was applied, and the treatment was maintained every 4 days to ensure the animal immunosuppression [23]. The mice survival was evaluated during 30 days post-infection. Animals that survive were immediately anaesthetized with 80 mg/kg ketamine and 10 mg/kg of xylazine (both from União Química Farmacêutica, Brazil) and then euthanized by cervical dislocation. Thus, the total experimental model duration was 34 days.

When the survival assay was carried out, rigorous monitoring of infected animals was done twice per day in order to detect any signs of advanced disease, pain or distress. Twenty six mice presented some of these symptoms and they were immediately euthanized to avoid any type of suffering, following the guidelines of the Guide for the Care and use of Laboratory animals. Ten animals died during the night with a rapid evolution of the disease, in this case, the mice were removed early the next day in order to avoid the stress of the other animals.

### 2.10. Biofilm Formation

The capacity to form biofilm by the yeasts was determined according to the protocol of Pierce and collaborators [24]. The yeasts of *C. auris* (Ca386, Ca432, Ca446, Ca881 and Ca885), *C. haemulonii* (LIPCh1, LIPCh11 and LIPCh3) and *C. albicans* (SC5314) as a control, were incubated overnight in Sabouraud dextrose broth (Becton, Dickinson and Company; Sparks, NV, USA) at 150 rpm and 37 °C. After this time, the medium was centrifuged, washed twice with PBS, and the final pellet was resuspended in RPMI medium. The suspensions of *C. haemulonii*, *C. auris* and the positive group were adjusted a final density of 1 × 10^6^ cells/mL. Then, 100 µl of these suspensions was spread in 96-well polystyrene plates, with a minimum of 6 replicates for each fungus. The negative control was only RPMI medium. After incubation at 37 °C for 24 h, the medium was aspirated carefully (to avoid the biofilm disruption) and washed three times with PBS to remove planktonic cells. Once the wells were dry, a solution of 2,3-bis(2-metoxi-4-nitro-5sulfo-fenil)-2H-tetrazolium-5-carboxanilida (XTT, Sigma-Aldrich, Saint Louis, MO, USA) containing menadione was added and incubated for 2 h. Finally, 75 µl of the supernatants were transferred into a new plate and the absorbance at 490 nm was measured in spectrophotometry.

### 2.11. Statistical Analysis

Statistics were performed using GraphPad Prism version 6.0 (GraphPad Software, San Diego, CA, USA). Statistical comparisons were made by analysis of variance (one-way ANOVA) followed by a Tukey–Kramer post hoc test. *p*-values of ˂0.05 indicated statistical significance. In the case of the survival curve, the Log-rank (Mantel–Cox) test with *p*-values of ≤ 0.001 was used to indicate statistical significance. A 95% confidence interval was determined in all experiments.

## 3. Results

### 3.1. Galleria mellonella Infected with C. auris Survive Less than the Larvae Infected with the C. haemulonii Complex.

**Figure 1:**
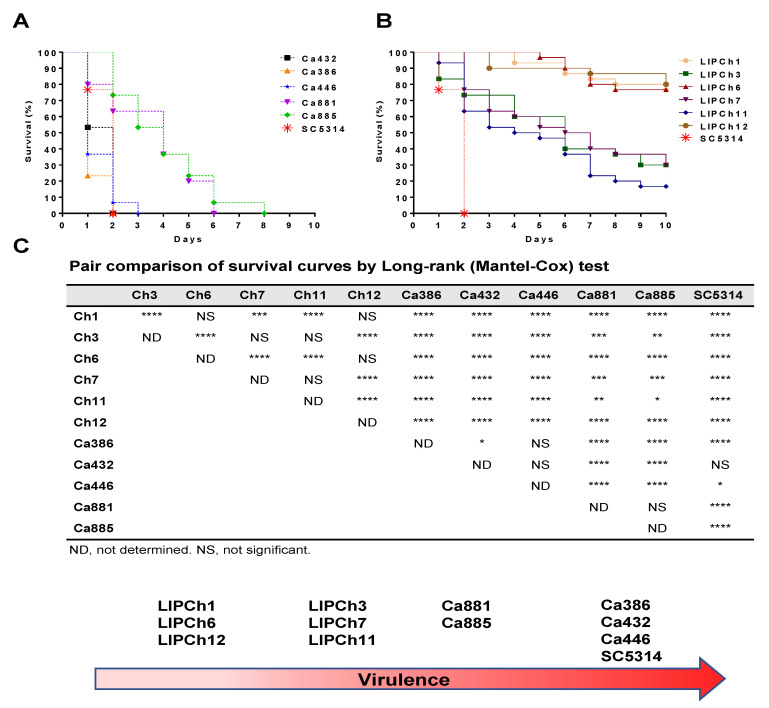
Survival curves of *Galleria mellonella* infected with 5 × 10^4^ yeasts of (**A**) *C. auris* (Ca432; Ca386; Ca446; Ca881 and Ca885) or (**B**) *C. haemulonii* species complex (LIPCh1; LIPCh3; LIPCh6; LIPCh7; LIPCh11; LIPCh12). Survival was measured for 10 days. *C. albicans* SC5314 was used as control group; (**C**) statistical comparison of each survival curve and subsequently classification in 4 virulence groups. Data are representative of three independent experiments. Long-rank (Mantel–Cox) test, *p* < 0.05. * *p* < 0.02, ** *p* < 0.001, *** *p* < 0.0005 and *p* **** *p* < 0.0001.

*G. mellonella* larvae showed a different survival profile depending on the fungal strain with they were infected (Figure 1A). *C. auris* strains Ca432, Ca386 and Ca446 together with *C. albicans* SC5314, used as a control strain, showed a significantly higher virulence than the remaining tested strains (Figure 1B—Group 1). The *C. auris* strains that showed lowest virulence were Ca881 and Ca885 (Figure 1B—Group 2). It is important to note that the two less virulent strains of *C. auris* were more lethal than the strains with greater virulence of *C. haemulonii* species complex. Regarding the strains belonging to the *C. haemulonii* complex, the most pathogenic ones were LIPCh3 and LIPCh7 (*C. haemulonii*) as well as LIPCh11 (*C. haemulonii* var. *vulnera*) (Figure 1B—Group 3), while the strains with the lowest virulence capability were LIPCh1 and LIPCh6 belonging to *C. duobushaemulonii* and LIPCh12 belonging to *C. haemulonii* (Figure 1B—Group 4). The majority strains forming the *C. haemulonii* complex showed low virulence in relation to the *C. auris* strains with an approximate larvae survival of 30% and in some cases 80% of survival.

### 3.2. Galleria mellonella Infected with C. auris Displayed a High Fungal Burden

**Figure 2:**
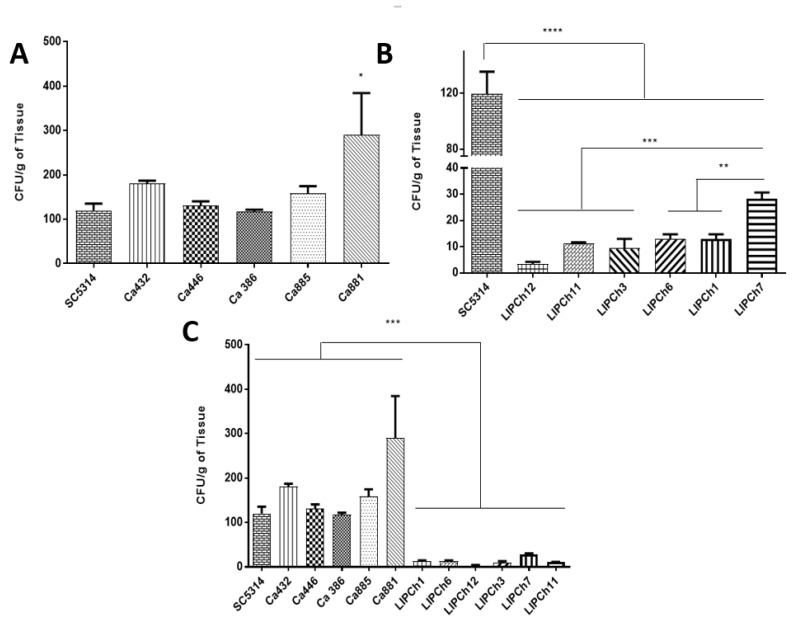
*Galleria mellonella* larvae were infected with 5 × 10^4^ yeasts of *C. auris* (Ca432; Ca446; Ca386; Ca885; Ca881) or *C. haemulonii* complex (LIPCh1; LIPCh6; LIPCh12; LIPCh3; LIPCh7; LIPCh11) and sacrificed 3 h or 3 days after infection, respectively. The colony forming units (CFUs) were quantified after 24 h of growth in Sabouraud agar plates. (**A**) CFU of larvae infected with different strains of *C. auris.* (**B**) CFU of larvae infected with different strains of *C. haemulonii* complex. (**C**) Comparison among all species tested. As positive control *C. albicans* SC5314 was used. Data are representative of three independent experiments. * *p* < 0.05, ** *p* < 0.001 and *** *p* < 0.0001. Error bars represent standard deviations between samples (biologic replicates) from the same group.

The number of CFU considering the larvae infected with different strains of *Candida* species showed a significant interspecific difference. In some cases, the CFU from larvae infected with *C. auris* was similar or even higher than the control group (*C. albicans* SC5314) as is the case of the larvae infected with Ca881 that presented the highest number of CFU compared with the other strains of *C. auris* and *C. albicans* (Figure 2A). It is important to note that there was a difference between the fungal burden of larvae infected with the different species of the *C. haemulonii* complex. Comparing the pathogenicity of the three species that integrate the *C. haemulonii* complex, the larvae infected with *C. haemulonii* (LIPCh7) presented the highest fungal burden compared with the others infected with *C. duobushaemulonii* (LIPCh1 and LIPCh6), *C. haemulonii*. var. *vulnera* (LIPCh11) and *C. haemulonii* (LIPCh3) that presented an intermediate fungal burden (Figure 2B). Within the *C. haemulonii* complex, the species that presented the lowest fungal burden was *C. haemulonii* (LIPCh12). The fungal burden of larvae infected with the species forming the *C. haemulonii* complex was significantly lower compared to larvae that were infected with the control strain of *C. albicans* and the five strains of *C. auris* (Figure 2C).

### 3.3. Larvae Infected with C. auris Leads to a Severe Tissue Invasion and Absence of Granuloma-Like Cell Aggregates

**Figure 3:**
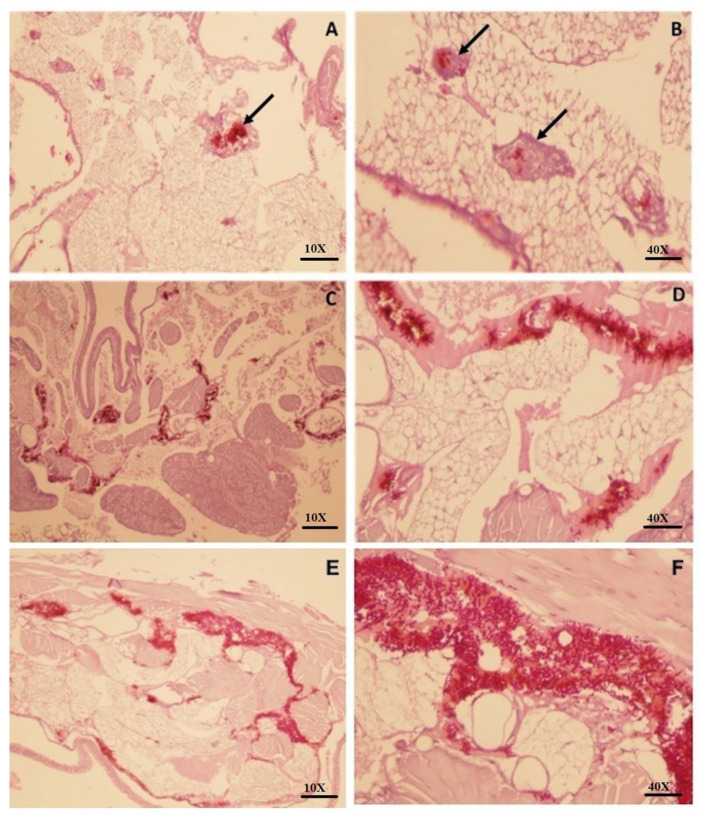
*Galleria mellonella* larvae were infected with 5 × 10^4^ yeasts of *C. auris* and *C. haemulonii* complex and sacrificed 3 h or 3 days after infection, respectively. The tissue was preserved in formalin and then stained with periodic acid–Schiff (PAS). (**A**) Larvae infected with *C. haemulonii* complex 10×; (**B**) larvae infected with *C. haemulonii* complex 40×; (**C**) larvae infected with *C. auris* (432) 10×; (**D**) larvae infected with *C. auris* (432) 40×; (**E**) larvae infected with *C. auris* (386) 10×; (**F**) larvae infected with *C. auris* (386) 40×. Black arrows show granuloma-like cell aggregates in the tissue.

Larvae infected with the *C. auris* (Ca432 and Ca386) strain showed a greater number of dispersed yeasts in the tissue, revealing a severe infection and invasion profile. At the same time, the formation of cellular aggregates was not observed. It is important to note that these two strains (Ca432 and Ca386) of *C. auris* did not form hyphae or pseudohyphae as it can be observed in the other strains of *C. auris* (Figure 3C–F). Contrarily, larvae infected with the most virulent species of the *C. haemulonii* complex (*C. haemulonii* (LIPCh3 and LIPCh7) and *C. haemulonii* var. *vulnera* (LIPCh11) with 3 days of infection showed a more controlled infiltration and well-defined formation of granuloma-like cell aggregates (black arrows) that could control the infection process (Figure 3A,B).

### 3.4. Virulence of C. auris and C. haemulonii in G. mellonella Model is Associated with Decrease in Phagocytosis

**Figure 4:**
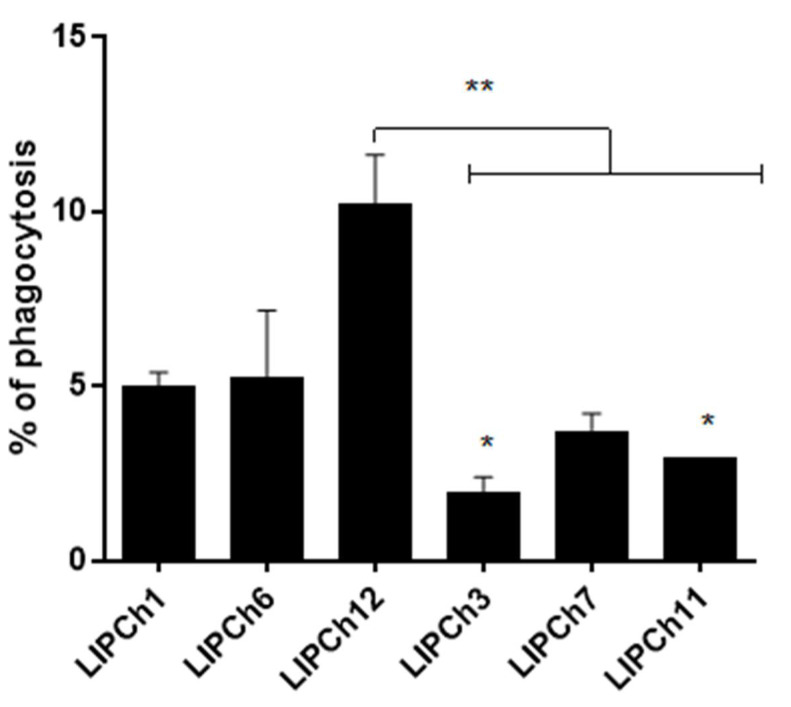
Larvae were infected with 5 × 10^4^ cells of *C. haemulonii* complex (LIPCh1; LIPCh6; LIPCh12; LIPCh3; LIPCh7; LIPCh11) and after 3 days of infection the hemolymph was extracted. A smear in glass slide was prepared and stained with Wright dye. Per group were used 100 hemocytes to obtain the phagocytic percentage. ** Indicates statistical significance ANOVA with post-Tukey’s test, *p* < 0.05 and * Indicates statistical significance *t*-test compared to *C. haemulonii* with low virulence (Ch1 and Ch6). Error bars represent standard deviations between samples (biologic replicates) from the same group.

In the phagocytosis assay, we observed that *G. mellonella* larvae infected with the most virulent strains of *C. auris* (432 and 386) showed a significant decrease in hemocytes and remarkable cell lysis compared with larvae infected with *C. haemulonii* complex or those that received less virulent strains. Another important point is that larvae infected with the most virulent species of the *C. haemulonii* complex (LIPCh3, LIPCh7 and LIPCh11) presented a significant decrease in phagocytosis when compared with the less virulent strains of the same fungal species complex (LIPCh1, LIPCh6 and LIPCh12) (Figure 4). Larvae infected with the species of the *C. haemulonii* complex showed no significant decrease in hemocyte density and no cell lysis as observed in tests carried out with different strains of *C. auris*.

### 3.5. (C) auris and C. haemulonii Complex Infection have the Same Mortality Profile in an Immunosuppressed Animal Model

**Figure 5:**
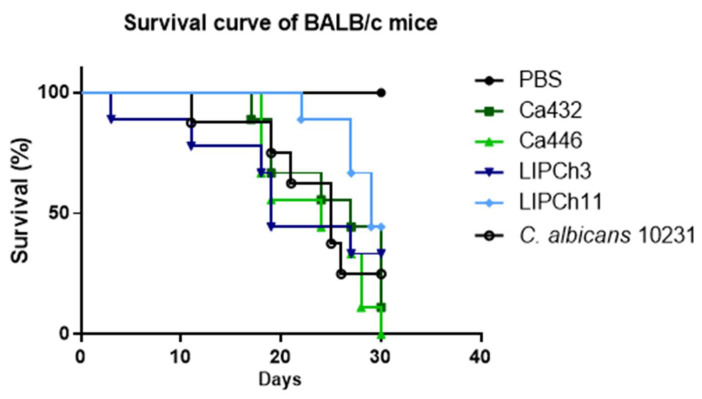
Animals (*n* = 10/group) were immunosuppressed with cyclophosphamide and infected with 10^4^ yeasts of *C. auris* (Ca432 and Ca446), *C. haemulonii* complex (LIPCh3 and LIPCh11) or *C. albicans* 10,231 (control group). Animal survival was observed for 30 days after infection. Significance compared to PBS-inoculated group (Long-rank test, *p* < 0.05). Data are representative of three independent experiments.

In the murine experimental model, observed for 30 consecutively days, no statistically significant difference in mortality was found between mice infected with *C. auris* (432 and 446) or with the species of the *C. haemulonii* complex (LIPCh3 and LIPCh11). However, up to the 30th day of infection, the mice mortality infected with *C. auris* (Ca446) was 100%. Animals infected with *C. auris* (Ca432) presented a mortality similar (80%) to those infected with the control strain of *C. albicans* ATCC10231. Female BALB/c mice that were inoculated with *C. haemulonii* (LIPCh3) and *C. haemulonii* var. *vulnera* (LIPCh11) had a survival close to 50% (Figure 5).

### 3.6. Biofilm Formation by C. auris and C. haemulonii Complex

**Figure 6:**
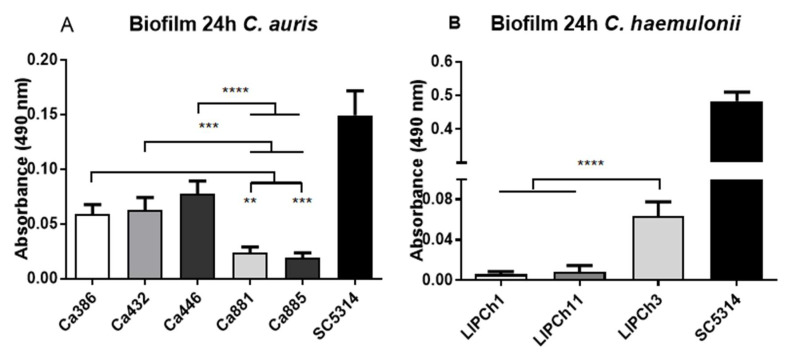
*C. auris* (**A**) and *C. haemulonii* (**B**) isolates were incubated in RPMI medium and after 24 h, the biofilm formation was revelated by XTT/menadione addition. The colorimetric reaction was read spectrophotometrically (490 nm). Statistic differences were observed between the isolates of high and low virulence in both experiments. *C. albicans* SC5314 was used as positive control. ** *p* < 0.05, *** *p* < 0.001, **** *p* < 0.0001. Error bars represent standard deviations between samples (biologic replicates) from the same group.

The virulent *C. auris* isolates Ca386 and Ca432 presented the greatest ability to generate biofilm compared to those less virulent as Ca881 and Ca885 (Figure 6A). In relation to the species of the *C. haemulonii* complex, LIPCh3 strain showed the highest metabolic activity in the biofilm formation when compared with the lowest virulent species LIPCh1 from *C. duobushaemulonii*. Similarly, the LIPCh11 strain of the *C. haemulonii* complex presented a low biofilm viability (Figure 6B). Visually, some differences in the biofilm appearance formed by the species of the *C. haemulonii* complex were observed. The biofilm produced by the *C. haemulonii* complex was thinner than those formed by *C. auris*. In addition, biofilm metabolic activity differences were observed in *C. auris* and the species of the *C. haemulonii* complex (see the Y axis in Figure 6A,B). In both experiments, *C. albicans* SC5314 was used as positive control due to its capacity to form a very highly heterogeneous biofilm.

## 4. Discussion

In the present work, we evaluated the pathogenic capacity of cryptic species of the genus *Candida* using both in vitro and in vivo approaches. One of them is *C. auris,* recognized for its resistance and persistence on the surfaces and the other yeasts belong to the *C. haemulonii* complex (*C. haemulonii, C. haemulonii* var. *vulnera* and *C. duobushaemulonii*), which are phylogenetically related to *C. auris*.

As observed in this study, the survival of *C. auris* infected larvae was relatively short compared to larvae infected with the species of the *C. haemulonii* complex, suggesting that the *C. auris* strains utilized in this survey are more pathogenic than those from the *C. haemulonii* complex. Similar results were obtained by Gandra et al. (2020) [25], who described that *C. haemulonii* was less virulent than *C. albicans* in the *G. mellonella* model. Moreover, in that same work, it was described that to obtain comparable mortality rates between these two species, it was necessary to use an inoculum ten times larger of *C. haemulonii* yeasts [25]. On the other hand, the survival curve of *G. mellonella* larvae, infected with some strains of *C. auris* showed a high pathogenicity that was very similar to that observed in larvae infected with the *C. albicans* strain used as positive control. In the same way, Sherry and coworkers (2017), using the *G. mellonella* model, found that non-aggregating *C. auris* strains were more virulent than some *C. albicans* strains tested [26].

Regarding the fungal burden in the experimental model of *G. mellonella*, it is important to note that the growth of *C. auris* was very similar to the strain of *C. albicans* used as positive control; C *auris* (Ca881) presented the major fungal burden of all *C. auris* strains and the positive control (SC5314). However, in the survival assay, the Ca881 strain was located within the second group of more virulent strains (Ca881 and Ca885) causing 100% mortality on the sixth day of infection. Results of the fungal burden assay indicated that despite the high concentration of CFU produced by the Ca881 strain, other strains such as Ca386, Ca446 and Ca432 are more virulent and caused earlier mortality of infected larvae. A previous study, using strains of *C. auris* obtained in medical centers in the United Kingdom, indicated that some strains of this species may present a virulence significantly greater than others in terms of the kinetics of larval death and the number of dead larvae, mainly when *C. auris* does not form cellular aggregates [27]. Our results strongly suggest that the *C. auris* strains utilized are more virulent in the invertebrate model of *G. mellonella* than those from the *C. haemulonii* complex (Figure 1).

Histopathology of *G. mellonella* larvae infected with *C. auris* and the species of the *C. haemulonii* complex showed a more aggressive infection process into the tissues of the larvae exposed to *C. auris.* Granuloma-like formation was a constant in the histopathology of larvae exposed to *C. haemulonii* complex strains (arrows Figure 3) probably due to the reduced evasion capacity of these yeasts against larvae immune responses corroborated by a reduced fungal burden and the larvae survival prolongation. As we noted at the moment to grow the strains, *C. auris* duplicated after two hours, while yeasts of the *C. haemulonii* complex duplicated after three hours. Probably, the growth rate also is related to the virulence profile exhibit for the yeasts analyzed. An interesting feature is that the formation of pseudohyphae was not observed in all the *Candida* species tested herein. In the case of *C. auris*, some authors highlight the inability of this species to produce pseudohyphae, germ tube, chlamydoconidia and chlamydospores [28]. On the other hand, different authors have described that the formation of a rudimentary and occasional pseudohyphae may occur, specific to some strains or under certain conditions, as stress response [29]. The formation of hyphae, pseudohyphae and germ tube in other yeast species such as *C. albicans* and *C. tropicalis* has been associated with high virulence [30]. In the near future, it will be important to deeply analyze the virulence factors of *C. auris* that could avoid the larvae immune system and that would be absent in the *C. haemulonii* complex strains.

The phagocytosis plays an important role in the innate immune response of *G. mellonella*. Hemocytes represent the main element of the cellular response of these larvae. In our study, we observed a drastic decrease in the number of hemocytes of larvae infected with *C. auris* due to a generalized lysis that does not allow us to carry out the phagocytosis rate analysis for all *C. auris* strains tested. This lysis profile of hemocytes observed in the case of *C. auris* is probably related to the possible virulence factors produced by this fungus that destroy the larval hemocytes. In the same way, we suggest that *C. haemulonii* species complex also have virulence factors that protect it from being totally phagocyted, but there are not as efficient as those of *C. auris*. The hemocyte lysis observed also can be related to the high and rapid death rates of the larvae with only two days post-infection that we noticed in the survival test. Previous works had shown that there is a close relationship between the number of hemocytes and the larvae survival rate, where larvae inoculated with high pathogenic strains have a reduced density of hemocytes, while those inoculated with low pathogenic strains show only a small variation in their hemocytes density [31,32]. In the case of larvae infected with the species of the *C. haemulonii* complex, was possible to carry out the experiment. We observed the lowest percentage of phagocytosis in the larvae infected with the most virulent strains of the *C. haemulonii* complex as LIPCh3, LIPCh7 and *C. haemulonii* var. *vulnera* (LIPCh11), probably due to the hemocyte decrease or to the production of virulence factors such as hydrolytic enzymes etc. However, it is important to note that phagocytosis can already be observed within a few hours after infection with this *Candida* species as referred by Silva et al. 2018 [33].

To study the disseminated candidiasis model, we used immunosuppressed BALB/c mice with the aim of better understand the pathogenicity processes of the different *Candida* species analyzed. Why we use immunosuppressed mice is that it allows us to better observe the differences in pathogenicity of the yeasts, since mice with a normal immune system could resolve the infection on their own or it would be necessary to use a very concentrated inoculum to observe differences in the infection process. We utilized the intravenous challenge model of disseminated infection that is commonly used to determine the virulence of *C. albicans* strains [34]. When we observed the virulence of *C. auris* and the species of the *C. haemulonii* complex in the murine experimental model, there was no significant difference in the survival of the mice infected with the yeasts tested. Some authors have described the poor ability of *C. auris* to infect and spread in mice compared with other *Candida* species [35]. However, Ben-Ami et al. (2017) [30] suggested a high virulence and pathogenicity of *C. auris* in mice, but specifically using strains that form cellular aggregates. Other studies highlighted the virulence of *C. auris* compared to other species such as *C. albicans* and *C. glabrata* in the immunocompetent murine model [36]. After all, we believe it is necessary to carry out more specific studies in more experimental models that could help us to better understand the process of infection and colonization of *Candida auris* that becomes more frequent and resistant.

Another important characteristic in the pathogenicity of *Candida* is the ability to form biofilm, which protects the yeasts from the action of different cells of the immune system—as well as the action of antifungals. When we analyzed the capacity of biofilm formation of the two species here studied by the XTT assay, we suggest that *C. auris* has a greater capacity to form viable biofilm than the species of the *C. haemulonii* complex. However, more specific experiments as the crystal violet test could be done in a near future to strengthen these results. Unexpectedly, the species *C. haemulonii* var. *vulnera* LIPCh11 did not exhibit the same biofilm behavior as LIPCh3 despite being also a highly virulent species in relation with the fungal burden and the larvae mortality. Our results corroborate what was found and described by Sherry and collaborators in 2017 [26]. However, it is important to note that these two species of *Candida* presented a lower biofilm production compared with the positive control (*C. albicans* SC5314). Hence, that, in the case of the strains of *C. auris* and *C. haemulonii* complex herein analyzed, the biofilm formation seems not to be a remarkable pathogenic characteristic.

In summary, the strains of *C. auris* present a significantly higher virulence compared to the species belonging to the *C. haemulonii* complex used in the experimental model of *G. mellonella*. However, in the murine model the difference in virulence of these fungal species was not as marked. However, the evasion of phagocytosis, as well as the biofilm formation of the *C. auris* strains, undoubtedly present important virulence factors that make this yeast an agent more adapted to the infection process in the host.

## Figures and Tables

**Table 1:** Identification and antifungal susceptibility profiles of the *C. haemulonii* complex and *C. auris* strains included in this study.

Strain	Isolation Date	Source	Country	Susceptibility Profile	
				AMB	FLZ
***C. auris* 432**	**2014**	**Craniotomy (Secretion)**	Colombia	R	R
*C. auris* 446	2015	Blood culture	Colombia	S	R
*C. auris* 386	2015	Bone Tissue (Biopsy)	Colombia	S	R
*C. auris* 885	2016	Blood culture	Colombia	S	R
*C. auris* 881	2016	Cerebrospinal fluid	Colombia	S	R
*C. duobushaemulonii* LIPCh1	2005	Finger nail	Brazil	R	R
*C. duobushaemulonii* LIPCh6	2009	Toe nail	Brazil	R	R
*C. haemulonii* LIPCh3	2009	Toe nail	Brazil	R	R
*C. haemulonii* LIPCh7	2009	Toe nail	Brazil	R	R
*C. haemulonii* LIPCh12	2013	Blood	Brazil	R	R
*C. haemulonii* var. *vulnera* LIPCh11	2013	Blood	Brazil	R	R

R—resistant; S—susceptibility; AMB—amphotericin B; FLZ—fluconazole.
